# Changes in Physiological Levels of Cortisol and Adrenocorticotropic Hormone upon Hospitalization Can Predict SARS-CoV-2 Mortality: A Cohort Study

**DOI:** 10.1155/2022/4280691

**Published:** 2022-02-25

**Authors:** Iraj Ahmadi, Hamideh Estabraghnia Babaki, Maryam Maleki, Hashem Jarineshin, Mohammad Reza Kaffashian, Mehdi Hassaniazad, Azra Kenarkoohi, Amin Ghanbarnejad, Shahab Falahi, Mitra Kazemi Jahromi, Hori Ghaneialvar, Shahla Sohrabipour

**Affiliations:** ^1^Department of Physiology, Faculty of Medicine, Ilam University of Medical Sciences, Ilam, Iran; ^2^Non-Communicable Diseases Research Center, Ilam University of Medical Sciences, Ilam, Iran; ^3^Anesthesiology, Critical Care and Pain Management Research Center, Hormozgan University of Medical Sciences, Bandar Abbas, Iran; ^4^Infectious and Tropical Diseases Research Center, Hormozgan Health Institute, Hormozgan University of Medical Sciences, Bandar Abbas, Iran; ^5^Department of Microbiology, Faculty of Medicine, Ilam University of Medical Sciences, Ilam, Iran; ^6^Social Determinants in Health Promotion Research Center, Hormozgan Health Institute, Hormozgan University of Medical Sciences, Bandar Abbas, Iran; ^7^Zoonotic Diseases Research Center, Ilam University of Medical Sciences, Ilam, Iran; ^8^Endocrinology and Metabolism Research Center, Hormozgan University of Medical Sciences, Bandar Abbas, Iran; ^9^Biotechnology and Medicinal Plants Research Center, Ilam University of Medical Sciences, Ilam, Iran

## Abstract

There is some indication that coronavirus disease 2019 (COVID-19) causes hypothalamic-pituitary-adrenal axis insufficiency. However, being on glucocorticoids makes it difficult to fully investigate this axis, especially in patients with severe COVID-19. We aimed to discover if there was a connection between blood total cortisol and adrenocorticotropic hormone (ACTH) levels and mortality in patients with COVID-19. In Iran, 154 hospitalized patients with COVID-19 were studied in a prospective cohort study. ACTH and cortisol levels in the blood were measured on the first or second day of hospitalization. Most patients (52.6 vs. 47.4%) were men over 50 years old (55.8%), and 44.4% had an underlying illness. Serum cortisol and plasma ACTH medians were 15.6 (*μ*g/dl) and 11.4 (pg/ml), respectively. 9.09% of the patients died. Cortisol levels were substantially lower in those who died (11.3 *μ*g/dl) than in patients who were discharged (16.7 *μ*g/dl, *P* < 0.01), while ACTH levels were unaffected. The most important factors determining mortality, according to the logistic model, were blood cortisol levels, the existence of an underlying disease, and the use of a mechanical ventilator. Cortisol levels that rose by one-unit correlated with a 26% lower risk of mortality. Comorbidities and mechanical ventilation increased the risk of death by 260 and 92 times, respectively. It can be concluded that in patients with COVID-19, a low cortisol level is linked to a high risk of mortality. Patients may sometimes have relative primary adrenal insufficiency. To judge and decide on therapeutic interventions, more reliable and long-term follow-up studies are required.

## 1. Introduction

Cortisol is released in a typical diurnal schedule in stress-free settings, with the highest peak in the early morning and a low amount in the evening. Any stress, including news of a pandemic, causes severe stress in people with coronavirus disease 2019 (COVID-19) infection. Increased serum cortisol is an adaptive survival mechanism to stimulate the immune and cardiovascular systems to overcome this stress via the hypothalamus-pituitary-adrenal axis [1]. Few studies have looked at the impact of COVID-19 infection on cortisol and adrenocorticotropic hormone (ACTH) levels in the blood. In two investigations, researchers found that blood cortisol levels were higher in patients with COVID-19 compared with COVID-19-negative individuals [1, 2]. Güven and Gültekin measured blood cortisol levels upon admission to the intensive care unit (ICU) [1], while Tan et al. measured them in the first 48 hours following hospital admission [2]. The death rate increased by 42% when cortisol levels were doubled. This indicates the severity of the condition [2]. Cortisol and ACTH levels were tested and repeated numerous times in patients with positive COVID-19 in another investigation. Cortisol levels dropped in certain patients, leading to the diagnosis of adrenal insufficiency [3]. Some researchers suggest that patients with COVID-19 may have developed adrenal insufficiency as a result of the coronavirus [3–5]. Adrenal microinfarctions and lesions have been found in the autopsy of some patients with COVID-19 [4–8]. However, further animal and in vitro research is needed on this topic. In studies on patients with SARS-CoV (very similar toSARS-CoV-2), secondary hypocortisolism and low ACTH levels were identified in a number of patients even after recovery [8]. Although hypotension is common in patients with COVID-19 [9], there have been few studies that have shown acute adrenal insufficiency, and more research is needed. The adrenal pituitary gland's hypothalamic axis determines how well the body functions and a poor response may increase mortality [1]. It is difficult to perform a complete evaluation of the hypothalamic-pituitary-adrenal axis, particularly in patients with COVID-19 who are heavily glucocorticoid dependent. We aimed to investigate the link between total blood cortisol and ACTH levels and the death rate among patients with COVID-19 upon hospital admission due to inconsistencies in previous research.

## 2. Methods

### 2.1. Study Design

This prospective cohort comprised 154 admitted patients with COVID-19 confirmed by real-time reverse transcription polymerase chain reaction (RT-PCR) at two large teaching hospitals in Bandar Abbas and Ilam (two pandemic hospitals in Iran). This research was carried out from March 15, 2020, to August 15, 2020. If they had not received corticosteroid therapy or had no previous history of taking corticosteroids for at least six months and no history of previously recognized pituitary or adrenal gland dysfunction, they were enrolled in the study. On the first or second day of admission, blood samples for serum cortisol and plasma ACTH levels were taken in the morning between 6:00 AM and 8:30 AM. On the same day, routine blood tests, CRP (c-reactive protein) level, erythrocyte sedimentation rate (ESR), D-dimer level, white blood cell (WBC) count, neutrophil and lymphocyte percentage, creatinine, blood glucose, sodium and potassium, vital signs, and pulse oximetry O2 saturation (SPO2) were collected from the patients' medical records. Moreover, ICU admission, inpatient days, oxygen therapy, invasive ventilation, and outcome were recorded.

The Cortisol AccuBind ELISA Kits (USA) were used to measure cortisol levels. ACTH was measured using a chemiluminescent technique on blood EDTA plasma (IMMULITE® 2000 ACTH, Munich, Germany).

### 2.2. Ethical Considerations

Written consent was obtained from each patient after a full explanation of the purpose and nature of all procedures used. The Hormozgan and Ilam University of Medical Sciences Ethics Committees approved the study (IR.HUMS.REC.1398.469 and IR.MEDILAM.REC.1399.332, respectively).

### 2.3. Statistical Analyses

SPSS software, version 24, was used to analyze the data (IBM, Chicago, Illinois, United States). The Kolmogorov–Smirnov test was used to determine the normality of quantitative variables. We utilized frequency (percent) and median (IQR: interquartile range) to describe categorical and continuous data, respectively. The categorical variables were analyzed using the chi-square test. The Mann–Whitney U test was used to compare continuous variables between the two groups. The link among the pairs of variables was investigated using Spearman's correlation test. The association between connected demographic and clinical parameters and patient death was investigated using logistic regression analysis.

## 3. Results

**Figure 1:**
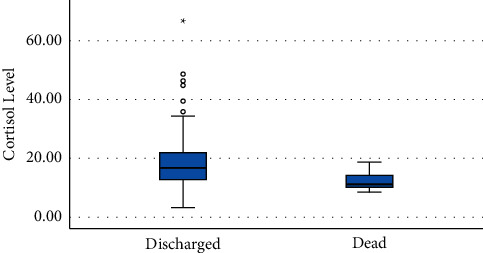
Distribution of cortisol levels in the two groups (discharged vs. dead patients).

154 patients in Bandar Abbas and Ilam who were sent to the reference hospital for COVID-19 were studied in this prospective cohort. All variables in the study were not normally distributed (*P* > 0.05) according to the normality test for continuous variables.  Table 1 lists the participants' baseline and demographic information. COVID-19 infection was seen in more men than in women (52.6% versus 47.4%), and most patients were over 50 years old. One or more underlying diseases afflicted 44.4% of the population. CRP was graded on a scale of one to four. One plus grade one, two plus grade two, three plus grade three, and more than four plus grade four were taken into account, with grade three being the highest CRP of our patients. The median levels of serum cortisol and plasma ACTH were (15.6 *μ*g/dl) (IQR: 11.6–21.2) and (11.4 pg/ml) (IQR: 5.8–18.0), respectively. Our patients' median ESR was higher than normal (30.0 mm/h, range: 13.0–50.0). SPO2 had a median of 91%, which was lower than the typical range (Table 1). The next step was to use the chi-square test to look into the relationship between categorical factors and outcome (0 = discharged and 1 = died). The results are shown in Table 2. The Mann–Whitney test was used to compare continuous variables, and the results are also shown in Table 2. 9.09% of the 154 patients died (five men and nine women). Mortality did not differ between men and women (*P*=0.19). Patients over 50 years (*P*=0.001), patients in the ICU (*P*=0.001), patients using an invasive ventilator (*P*=0.001), and patients with at least one underlying disorder (*P*=0.001) all had a high mortality rate. On the first day of admission, expired patients had a lower SPO2 percentage (92% versus 84.5%). Serum cortisol levels were substantially lower in expired patients (11.3 *μ*g/dl [9.9–14.3]) than in discharged patients (16.7 *μ*g/dl [12.5–21.7], *P* < 0.01), although there was no difference in plasma ACTH levels (pg/ml) between the two groups. Electrolytes of sodium and potassium were similar in deceased and living individuals on the first day of admission. The D-dimer level was substantially greater in deceased patients (177.7 vs. 750.0 ng/ml). The blood creatinine level was also higher. WBC count, neutrophil and lymphocyte percentage, neutrophil-lymphocyte ratio, ESR, and CRP, on the other hand, did not differ significantly. The logistic regression method was utilized to determine which factors had an impact on the death outcome. The variables that were significant at level 0.1, according to Table 2, were incorporated into the logistic model for this purpose. After controlling for other variables, serum cortisol level, having an underlying condition, and receiving mechanical ventilation were factors affecting patient death. As shown in Table 3, a one-unit increase in cortisol level was associated with a 26% reduction in the risk of death; a one-unit increase in D-dimer was associated with a 0.2% increase in the risk of death; patients with the underlying disease were about 260 times more likely to die than those without it; and using a mechanical ventilator increased the risk of death by approximately 92 times.  Figure 1 shows a box plot of cortisol levels in the two groups (discharged vs. died). The distribution of cortisol in nonsurvivors was more concentrated and was completely in the lower level, as shown in Figure 1. Among the deceased patients, 7 (50%) had hypotension upon admission and received norepinephrine.

## 4. Discussion

Even though numerous efforts were made to treat and save patients with COVID-19, mortality rates remained high [10]. Patients with COVID-19 had a mortality rate of about 10%, according to our and other previous studies [11, 12], compared to 59.61% for those admitted to the ICU [10].

The number of men with COVID-19 was more than that of women (52.6% vs. 47.4%), as in practically all previous studies, because of differing levels of angiotensin-converting enzyme 2 (ACE2) receptor in the two sexes and hormonal differences [11, 13–15]. Similar to other studies, we found that underlying diseases, advanced age, lower SPO2 levels on admission, increased blood levels of D-dimer and creatinine, ICU admission, and mechanical ventilation all contributed to the increased number of patients with COVID-19 mortality rate [11, 16–19]. Various types of stress can be dealt with by our body's ability to suppress them, which is provided through the hypothalamic-pituitary-adrenal axis's proper, accurate, and timely reactions [1]. Furthermore, such stresses can be alleviated by boosting blood cortisol levels and adjusting metabolic, cardiovascular, and immunological systems. Stress has the ability to reduce cortisol metabolism and cortisol-binding globulin (CBG) metabolism, resulting in increased cortisol function [2]. People were severely stressed by the announcement of the SARS-CoV-2 epidemic, which resulted in a spike in cortisol levels because of fear of death [1]. Unfortunately, there are few investigations on COVID-19's effects on the hypothalamic-pituitary-adrenal axis and blood cortisol levels.

In one study on probable COVID-19-infected cases with no symptoms of adrenal insufficiency and/or glucocorticoid therapy, baseline serum cortisol levels were measured within 48 hours of patients' admission. In their investigation, patients with COVID-19 infection had a median serum cortisol concentration of 22.43 *μ*g/dl compared to 18.81 *μ*g/dl in those who were not infected with COVID-19. Increased serum cortisol levels were the prognosis of acute mortality rates. Subjects with cortisol levels of less than 26.97 *μ*g/dl had a considerably higher survival rate than those with cortisol levels greater than 26.97 *μ*g/dl [2]. The researchers found that doubling cortisol levels increased mortality by 42% [2]. High cortisol levels at the time of admission may be related to the degree of systemic disease, which was linked to a lower rate of survival in patients with severe COVID-19. They obviously did not observe adrenal insufficiency in the acute phase of COVID-19 infection in this cohort analysis. They concluded that serum cortisol is a superior measure of sickness severity than CRP, D-dimer, and neutrophil-to-leukocyte ratio for COVID-19 infection [2]. The severity of the disease was not mentioned between the two groups. Because CRP was greater in patients with COVID-19 in this study, “which is a factor in the intensity of inflammation,” the severity of the condition may have been higher in patients with COVID-19. As a result, cortisol levels have been found to be higher in these patients [20]. The median amount of serum cortisol on the first day of ICU admission was 21.84 *μ*g/dl and 16.47 *μ*g/dl, respectively, in another study [1] between ICU-admitted patients with COVID-19 (confirmed by clinical and radiological characteristics rather than RT-PCR) and ICU-admitted patients without COVID-19, which was significantly different between the two groups. According to the authors, cortisol levels were lowest in living patients without COVID-19 and the greatest in deceased patients with COVID-19. Only the level of cortisol was statistically significant in the multivariate logistic regression analysis when compared to other laboratory tests. The cortisol cut-off limit was 31 *μ*g/dl, suggesting that it might be used as a marker to identify patients at risk of mortality and additional direct resources towards them [1]. Other laboratory tests such as CRP, creatinine, D-dimer, aspartate aminotransferase (AST), and neutrophil-to-leukocyte (N : L) ratio were higher in patients with COVID-19 than in patients without it admitted to the ICU in this study, indicating that the severity of the disease was greater in ICU-admitted patients with SARS-CoV-2, suggesting that these two groups are not comparable [1].

Cortisol, ACTH, and DHEAS (dehydroepiandrosterone sulfate) levels were assessed within 24–48 hours following the admission of only 28 COVID-19 positive individuals, most of whom were asymptomatic and/or had a moderate infection, in another small study [3]. The median values for cortisol, ACTH, and DHEAS were 7.1 *μ*g/dl (1.12–21.27), 18.5 ng/L (4–38), and 3 mol/L (0.27–11), respectively. These examinations were repeated with 20 patients over the next few days. Cortisol levels were found to be lower in seven patients who had been diagnosed with adrenal insufficiency. One patient had a single episode of hypoglycemia, while the other two had hypotension, both of which were symptoms of adrenal deficit. Cortisol and ACTH levels dropped during the next few days, while DHEAS levels remained within normal limits. Regarding the authors' conclusions, their patients had acquired secondary adrenal shortage. The previously mentioned study had two flaws: the study sample was limited, and the patients were asymptomatic or had mild COVID-19 infection. The authors stated that in vitro, clinical, and animal model investigations were needed to better study the occurrence of secondary adrenal deficit [3]. If cortisol and ACTH could be monitored in the future days, perhaps clearer results could be reached in our investigation as well. However, this was impossible because most patients began taking corticosteroids after the blood sample was taken. COVID-19-induced adrenal hemorrhage was reported in a few clinical cases, which can lead to adrenal insufficiency [4, 5], and in an autopsy case series, adrenal microinfarction and adrenal lesions were observed in the patients who died from COVID-19, indicating that hypoadrenalism can be fatal in some patients [6, 7]. Adrenal insufficiency diagnosis depends on the amount of blood protein. If blood albumin is normal (≥2.5 gr/dl), serum cortisol less than 10 *μ*g/dl is considered as adrenal insufficiency. Cortisol levels greater than 15 *μ*g/dl are considered normal. However, if the blood albumin level is less than 2.5 gr/dl, adrenal insufficiency is diagnosed when the serum cortisol level is less than 8 *μ*g/dl. A cosyntropin stimulation test is required between the minimal and normal levels to diagnose adrenal insufficiency [21]. Patients' albumin levels were not tested in most cortisol investigations, and patients were only acutely evaluated. The chronic effects of SARS-CoV-2 on the adrenal glands require more detailed, lengthier, and comprehensive research. Patients who survived SARS-CoV and had no preexisting endocrine abnormalities showed temporary hypocortisolism after recovery. Three months following recovery, nearly 40% of the patients had hypocortisolism, 83.3% of which had secondary hypocortisolism and low ACTH levels. Most of these individuals were not on exogenous glucocorticoids at the time of their sickness. The most common symptom among recovered individuals was orthostatic hypotension [22]. Although hypotension is common in patients with COVID-19 [9], and 22–67% of them use vasopressors [23], there are few studies showing acute adrenal insufficiency [24], and more research is needed. The blood pressure in our study was within normal limits on the day of blood sample collection, but at the time of hospitalization, 50% of our expired patients received vasopressor drugs, especially norepinephrine infusion. The physiological cortisol requirements may become unbalanced in situations such as physical and psychological stress [25]. Shock is typical in patients with SARS-CoV-2 who are very ill. It has been observed that up to 67% of ICU-admitted patients (5–10% overall) suffer from shock [26]. Most of these shocks were classified as sepsis, cytokine storm, and neurogenic, but some of them may have been caused by adrenal insufficiency [27]. In our study, adrenal insufficiency may appear to be of the primary type because the ACTH level did not decrease. As a coronavirus root entry, ACE2 has moderate levels of expression in the adrenal gland [28]. Autopsies of ten patients with COVID-19 revealed pathomorphological alterations in the adrenal glands, including the perivascular infiltration of CD3+ and CD8+T-lymphocytes [29]. Although ACTH levels did not change between the two groups in our investigation, the animals' hypothalamus and pituitary tissues had high ACE2, suggesting that the hypothalamus could be a viral infection target [30–32]. Biochemical evidence of hypothalamo-pituitary involvement in SARS was revealed by Leow and colleagues. SARS survivors showed signs of central hypocortisolism. Hypophysitis or a direct hypothalamic injury could have resulted in hypothalamo-pituitary dysfunction [22]. However, given SARS-CoV-2's frequency of neurological symptoms, it is reasonable to assume that SARS-CoV-2 may also affect the hypothalamus-pituitary system, either directly or via the immune-mediated hypophysitis [32]. Cortisol and ACTH tests were performed at the start of our study; perhaps in the coming days, further alterations will emerge, which have not been remeasured in most patients because of corticosteroid drugs. Along with ACTH (cosyntropin) stimulation tests, more comprehensive studies are required.

## 5. Conclusion

Although a previous study found that the death rate increased when at the time of the admission, cortisol levels were increased (2), our findings revealed that individuals with SARS-CoV-2 who had lower cortisol levels had a greater fatality rate. Later in the course of their illness, the patients may acquire some relative primary adrenal deficit. To summarize, further precise investigations with long-term follow-up are necessary.

## Figures and Tables

**Table 1:** Baseline characteristic of patients with COVID-19.

Variables	No. (%)	Median (interquartile range (IQR))
Gender		
Male	81 (52.6)	
Female	73 (47.4)	
Age category		
≤50 yrs	68 (44.2)	
>50 yrs	86 (55.8)	
Oxygen therapy		
No	18 (11.7)	
Yes	136 (88.3)	
Mechanical ventilator		
No	142 (92.8)	
Yes	11 (7.2)	
ICU administration		
No	120 (77.9)	
Yes	33 (21.4)	
C-reactive protein (qualitative; normal range 0)		
Negative	34 (22.5)	
1+	23 (15.2)	
2+	33 (21.9)	
3+	48 (31.8)	
4+	13 (8.6)	
Have underlying disease		
No	85 (55.6)	
Yes	68 (44.4)	
Age (years)		53 (40.0–65.3)
Serum cortisol level (*μ*g/dl)		15.6 (11.6–21.2)
Plasma ACTH level (pg/ml)		11.4 (5.8–18.0)
Systolic blood pressure (mmHg)		118.5 (110.0–130.0)
Diastolic blood pressure (mmHg)		75 (70.0–80.0)
Body temperature (°C)		37 (36.8–37.5)
SPO2 (%)		91.0 (88.0–95.0)
Hospitalized day		4.0 (3.0–6.0)
Sodium (mEq/L)		138 (136.0–141.0)
Potassium (mEq/L)		4.1 (3.8–4.3)
D-dimer (ng/ml)		200 (100.0–409.8)
Creatinine (mg/dl)		1.0 (0.9–1.2)
Glucose (mg/dl)		116.0 (98.0–149.0)
WBC (per mm^3^)		6100.0 (4800.0–7550.0)
Neutrophil (%)		75.0 (65.6–82.9)
Lymphocyte (%)		21.5 (15.0–29.6)
Erythrocyte sedimentation rate (mm/h)		30.0 (13.0–50.0)
Neutrophil-lymphocyte ratio (NLR)		3.3 (2.3–5.7)

**Table 2:** Comparison of demographic and clinical variables between discharged and expired patients.

Variables	Discharged (*n* = 140) no. (%)	Dead (*n* = 14) no. (%)	*P* value
Gender			
Male	76 (93.8)	5 (6.2)	0.19
Female	64 (87.7)	9 (12.3)	
Age (years)	51.5 (39.3–63.8)	70 (62.8–80.5)	<0.001
Age category			
≤50 yrs	68 (100)	0 (0)	<0.001
>50 yrs	72 (83.7)	14 (16.3)	
Oxygen therapy			
No	18 (100)	0 (0)	0.37
Yes	122 (89.7)	14 (10.3)	
SPO2%	92 (89.0–95.0)	84.5 (75.0–91.5)	<0.001
Having underlying disease			
No	83 (97.6)	2 (2.4)	0.001
Yes	56 (82.4)	12 (17.6)	
ICU administration			
No	118 (98.3)	2 (1.7)	<0.001
Yes	21 (63.6)	12 (36.4)	
Mechanical ventilator			
No	137 (96.5)	5 (3.5)	<0.001
Yes	2 (18.2)	9 (81.8)	
Body temperature (°C)	37.0 (36.70–37.50)	37.4 (36.9–37.9)	0.053
Systolic blood pressure (mmHg)	118.5 (110.0–130.0)	115 (108.8–150.0)	0.77
Diastolic blood pressure (mmHg)	75.0 (70.0–81.5)	77.5 (70.0–80.0)	0.68
Serum cortisol level (*μ*g/dl)	16.7 (12.5–21.7)	11.3 (9.9–14.3)	0.003
Plasma ACTH level (pg/ml)	11.8 (6.0–11.3)	9.1 (5.6–13.1)	0.30
Sodium (mEq/L)	139.0 (136.0–141.0)	137.0 (134.8–140.0)	0.12
Potassium (mEq/L)	4.1 (3.8–4.3)	4.0 (3.5–4.6)	0.98
D-dimer (ng/ml)	177.7 (100.0–379.0)	750.0 (200.0–1653.7)	<0.001
Serum creatinine (mg/dl)	1.0 (0.9–1.2)	1.4 (1.0–1.9)	0.003
Glucose (mg/dl)	115.0 (97.0–145.0)	137.0 (105.3–260.8)	0.09
WBC (per mm^3^)	6100 (4800–7500)	6900 (5200–14000)	0.23
Neutrophil%	73.8 (65.3–82.8)	78.7 (73.0–87.9)	0.14
Lymphocyte	22.4 (15.0–29.8)	16.0 (8.0–26.3)	0.10
Neutrophil-lymphocyte ratio (NLR)	3.3 (2.3–5.6)	5.0 (2.8–11.8)	0.09
Erythrocyte sedimentation rate (mm/h)	30.0 (14.0–50.0)	20.0 (10.0–39.0)	0.19
C-reactive protein (qualitative; normal range 0)			
Negative	33 (97.1)	1 (2.9)	0.46
1+	21 (91.3)	2 (8.7)	
2+	30 (90.9)	3 (9.1)	
3+	41 (85.4)	7 (14.6)	
4+	12 (92.3)	1 (7.7)	
Hospitalized day	4.0 (3.0–6.0)	5.0 (2.0–9.0)	0.50

**Table 3:** Multivariate logistic regression analysis on factors related to death among patients with COVID-19.

Variable	Coefficient	Wald statistics	Adjusted odds ratio (95% CI)	*P* value
Age	0.099	1.925	1.10 (0.96–1.27)	0.165
Serum cortisol level (*μ*g/dl)	−0.298	3.922	0.74 (0.55–0.99)	0.048^*∗*^
D-dimer (ng/ml)	0.002	6.353	1.002 (1.001–1.004)	0.012^*∗*^
Serum creatinine (mg/dl)	0.174	0.129	1.190 (0.46–3.08)	0.719
Body temperature (°C)	0.817	0.278	2.265 (0.11–47.16)	0.598
SPO2 (%)	−0.099	0.436	0.91 (0.67–1.22)	0.509
ICU administration	−0.415	0.071	0.66 (0.03–13.98)	0.790
Having underlying disease	5.560	3.860	259.80 (1.01–66604.06)	0.049^*∗*^
Mechanical ventilator	4.527	5.355	92.45 (2.00–4274.47)	0.021^*∗*^
NLR	0.122	0.360	1.13 (0.76–1.68)	0.549

CI = confidence interval; SPO2 = pulse oximetry O2 saturation; NLR = neutrophil-lymphocyte ratio; ICU: intensive care unit; ^*∗*^significant at level 0.05.

## Data Availability

The data used to support the findings of this study are available from the corresponding author upon request.
